# Canonical and hierarchical analyses of body measurements in four Nigerian cattle breeds under an extensive management system

**DOI:** 10.1007/s11250-025-04644-9

**Published:** 2025-11-13

**Authors:** J. J. Okoh, W. P. B. Putra, Y. Ibrahim, A. Maigado, S. Ibrahim, B. S. Ma’aruf, A. Shuaibu, J. P. Adakole, R. S. Harahap

**Affiliations:** 1 Department of Animal Science, Federal University of Kashere, Gombe, 771103 Nigeria; 2 Research Center for Applied Zoology, National Research and Innovation Agency (BRIN), Bogor, West Java 16911 Indonesia; 3 Department of Agricultural Science Education, Federal College of Education, Odugbo, Benue State 972107 Nigeria; 4 Department of Animal Production, Faculty of Animal Science, University of Jambi, Jambi, 36122 Indonesia

**Keywords:** Body measurement, Characterization, Nigerian cattle, CDA, HCA

## Abstract

Cattle are significant livestock worldwide, primarily kept for meat and milk production. Four Nigerian cattle breeds of White Fulani (Bos indicus), Gudali (Bos indicus), Azawak (Bos indicus), and Red Bororo (Bos taurus × Bos indicus) had the genetic potential to produce meat and milk optimally under extensive management systems. This study aimed to characterize these four adult Nigerian cattle breeds based on 26 body measurement variables. Two statistical analyses, canonical discriminant analysis (CDA) and hierarchical cluster analysis (HCA), were performed to differentiate the breeds using SPSS 16.0 software. The results revealed that 13 variables of head width, head length, body length, body width, body depth, chest length, chest girth, chest depth, rump length, rump width, hump length, hump width, and dewlap width were discriminant variables for characterizing the four Nigerian cattle breeds. Furthermore, the breeds Gudali (GU), Azawak (AZ), and White Fulani (WF) / Red Bororo (RB) could be classified into distinct clusters based on these morphometric traits. The canonical correlation coefficients were 0.95 for function 1 and 0.86 for function 2. However, the WF and RB breeds could not be discriminated based on their body measurements due to their close genetic distance (0.21). In contrast, the genetic distance between GU and AZ breeds was 1.77. Overall, the body measurements were able to classify approximately 87% of WF, 99% of GU, 98% of AZ, and 70% of RB into their original groups. In conclusion, the body measurements of WF and RB are similar, which may be attributed to their close genetic composition, likely resulting from continuous crossbreeding over several generations.

## Introduction

In Africa, and particularly in Nigeria, there exists a wide range of cattle breeds that represent a rich source of genetic diversity. However, these breeds have not been adequately studied or exploited. Among the notable cattle breeds in Nigeria are the Gudali, White Fulani, and Red Bororo. These breeds vary both physically and genetically within populations, with differences in morphology, physiology, and behavior observed between individuals, breeds, and populations (Frankham et al. 2002). Currently, genetic studies rely on phenotypic observations and biometric measurements, which focus on the description and measurement of gross morphology. Sometimes, these studies also encompass aspects of anatomy, physiology, and productivity (Pesmen and Yardimen 2008). The importance of phenotypic characterization has been emphasized by the Food and Agriculture Organization of the United Nations (FAO 2012), which recommends that the physical attributes included in such studies should be clearly defined, uniform, and universal. This ensures that comparisons can be made within and between breeds globally.

Before the advent of genetic studies, animal classification was based on historical and anthropological evidence (Mwacharo et al. 2006a, b). The White Fulani breed is the most widespread in Nigeria, accounting for 37% of the national herd. Pastoralists generally regard White Fulani cattle as superior to other Zebu breeds due to their strong resistance to diseases and their ability to thrive in diverse environmental conditions. However, this tropical breed faces certain limitations, such as late sexual maturity, long intervals between calving, and short lactation periods. Despite these challenges, the White Fulani is valued for its hardiness, heat tolerance, and adaptability to local conditions (Alphonsus et al. 2012).

The Red Bororo breed is the third most numerous cattle breeds in Nigeria, accounting for 22% of the national herd. It holds high prestige among Fulani pastoralists. The Gudali breed exists in two distinct types in Nigeria namely, Sokoto and Adamawa Gudali, originating from the Sokoto and Adamawa regions, respectively. The Sokoto Gudali accounts for approximately 30% of the national herd (NNLRS 1990; Alphonsus et al. 2012), while the less common Adamawa Gudali represents about 2% (Blench 1993). According to the 2025 Wikipedia entry on Adamawa and Sokoto Gudali cattle, these figures remain consistent, with the two breeds collectively representing around 32% of Nigeria’s cattle population. There is no current data indicating significant changes in these proportions. As such, the 2% and 30% estimates continue to be widely accepted as of 2024.

These three breeds (the Gudali, White Fulani, and Red Bororo) are well known for their quality meat and milk production, making them ideal subjects for further study on productivity and other traits.

In contrast, the Azawak cattle, which are highly localized and known for their hardiness, are primarily used as draught animals. Unfortunately, these cattle may never be fully studied for their potential, and their limited populations put them at risk of extinction. With so few individuals, indiscriminate mating with other breeds could lead to gene dilution, preventing a comprehensive study and characterization of the pure Azawak breed. Despite DNA mitochondrion analysis, the breeds characterization can be assessed with body measurements using canonical discriminant analysis (CDA) and hierarchical cluster analysis (HCA) techniques (Mustefa et al. 2024; Suryaka et al. 2024).

## Materials and methods

### Approval of ethics

The research follows ethical guidelines approved by the University’s Ethics Committee and IRB, involving animal subjects, and is free from plagiarism and data falsification. There is no conflict of interest, and the authors have contributed significantly to the study’s conception, design, analysis, and interpretation. The authors commit to upholding ethical principles to maintain scientific integrity.

### Management of animal

The animals were collected from cattle herders across the Northeastern region of Nigeria, where they are predominantly raised. These cattle are extensively grazed and moved from one location to another in search of pasture and water points. Data were collected on these animals at its resting points in several locations across Gombe, Bauchi, and Adamawa in Northeast Nigeria.

### Animals and research site

**Fig. 1:**
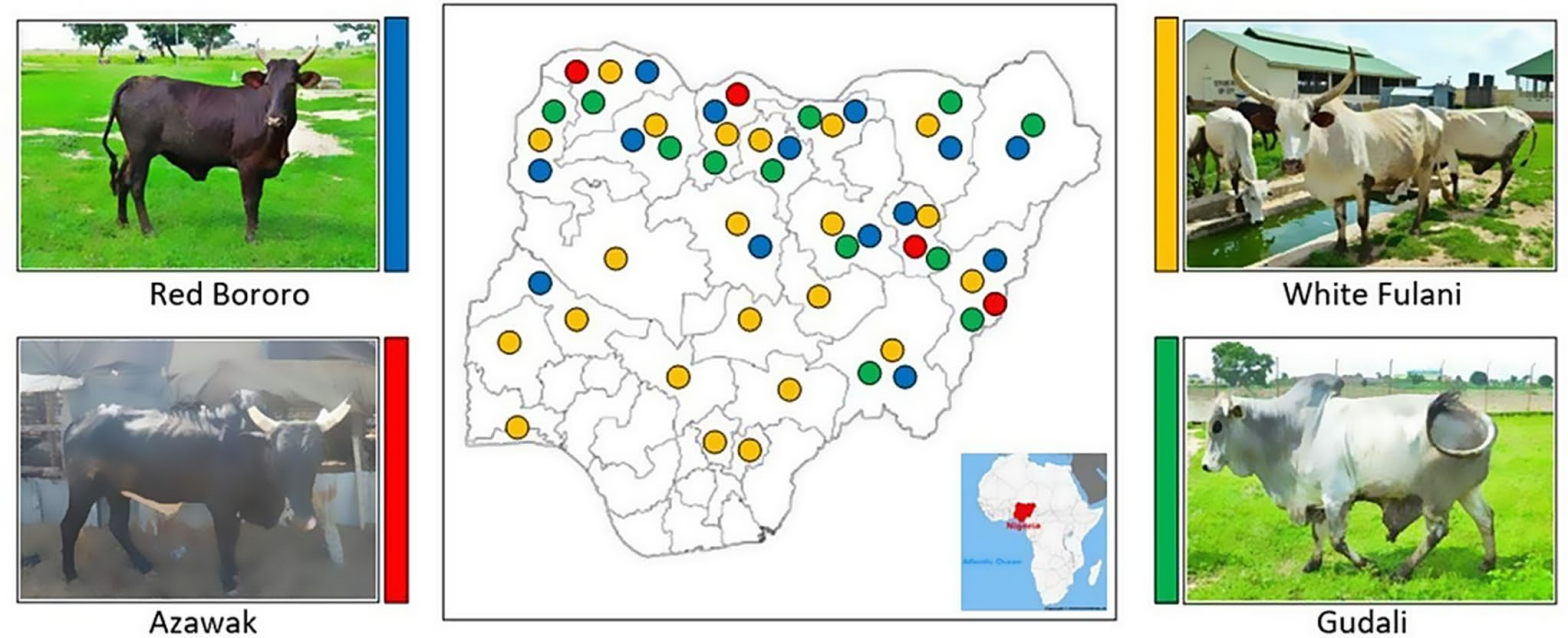
Phenotype characteristic and geographical distribution in four Nigerian cattle breeds of Red Bororo (blue), Azawak (red), White Fulani (yellow) and Gudali (green)

A total of 400 adult cattle, including White Fulani (WF), Gudali (GU), Azawak (AZ), and Red Bororo (RB) breeds, were used in the present study. Each breed were represented by 100 animals, with 50 males and 50 females. The phenotypic characteristics and geographical distribution of these breeds in Nigeria are illustrated in Fig. 1.

### Body measurements

A total of 26 body measurement parameters were recorded, including head width (HeW), head length (HeL), body length (BL), body width (BW), body depth (BD), chest length (CL), chest girth (CG), chest width (CW), chest depth (CD), sternum height (SH), withers height (WH), rump height (RH), rump length (RL), rump width (RW), hump length (HuL), hump width (HuW), horn length (HoL), ear length (EL), dewlap width (DW), tail length (TL), cannon bone circumference (CC), scrotal circumference (SC), scrotal length (SL), udder length (UL), udder diameter (UD), and udder teat length (UTL) in all samples of male and female animals, as described by Alderson (1999). Body measurements, such as height, heart girth, and length, were taken after the animal had been off feed and water for several hours. The animal was positioned with all four legs squarely under its body and its head held in a normal position. The measuring tape was then passed around the body just behind the shoulders, at the smallest circumference, and pulled reasonably snug.

**Fig. 2:**
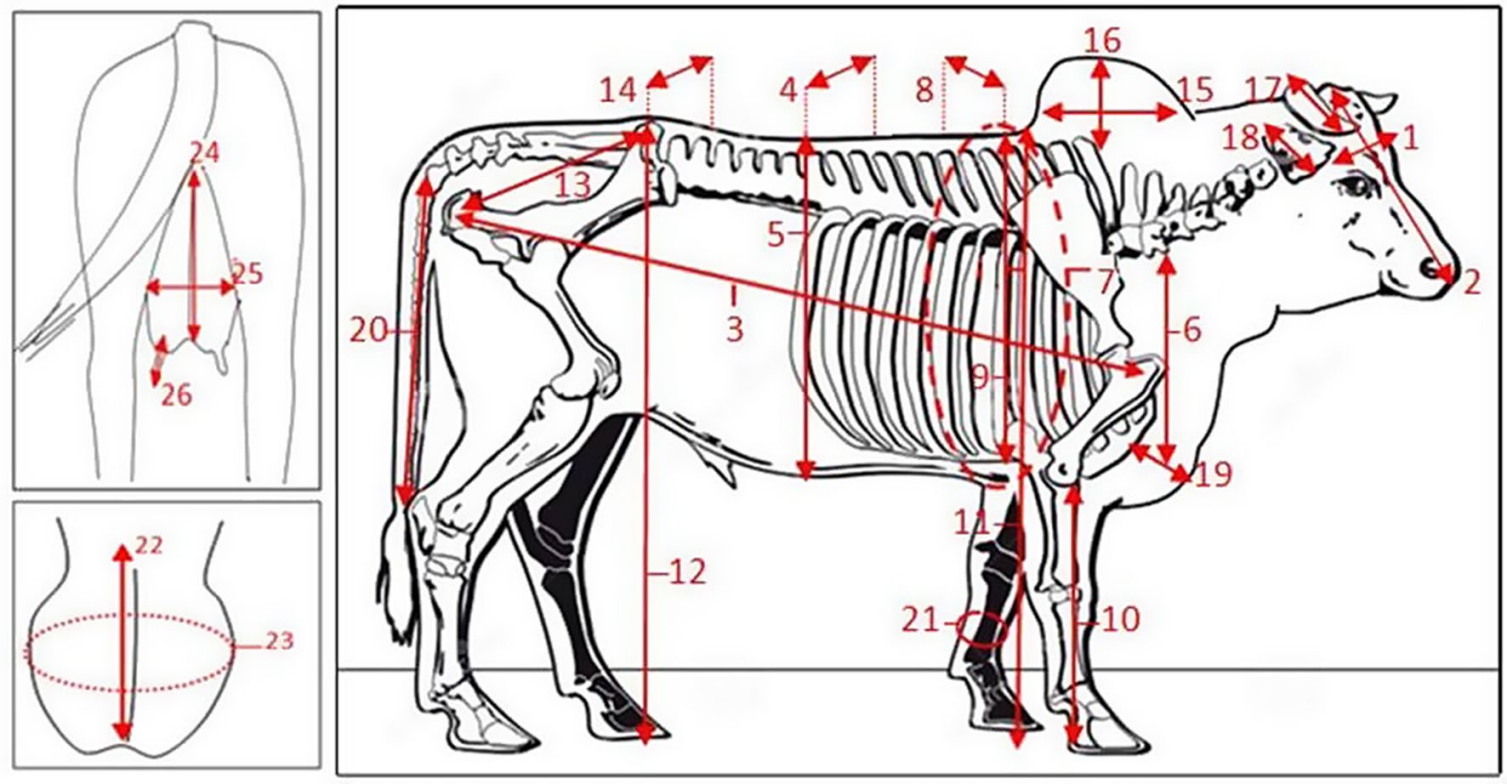
Scheme of body measurements in cattle for head width (1), head length (2), body length (3), body width (4), body depth (5), chest length (6), chest girth (7), chest width (8), chest depth (9), sternum height (10), withers height (11), rump height (12), rump length (13), rump width (14), hump length (15), hump width (16), horn length (17), ear length (18), dewlap width (19), tail length (20), cannon bone circumference (21), scrotal length (22), scrotal circumference (23), udder length (24), udder diameter (25) and udder teat length (26)

The weight was determined by wrapping the tape around the heart girth of the cattle, directly behind the elbow, and overlapping the ends of the tape. The resultant weight was read when the tape was snugly in place, at the time of respiratory expiration. A diagram of these measurements is shown in Fig. 2.

### Statistical analysis

The statistical parameters, including mean and standard deviation of body measurements, were calculated using SPSS 16.0 software. Two statistical analyses of Canonical Discriminant Analysis (CDA) and Hierarchical Cluster Analysis (HCA) were performed to characterize animals based on their body measurements. CDA was employed to classify observations into pre-existing groups (Asamoah-Boaheng and Sam 2016). In CDA, the Mahalanobis distance (D²), tolerance (T), Wilk’s lambda (λ), and linear discriminant function were computed to identify the discriminating variables among the four different cattle breeds. CDA was carried out using the backward stepwise automatic elimination method, with an entry F value of 3.84 and a removal F value of 2.71. The T value (ranging from 0 to 1) was used to assess the level of correlation between variables in the discriminant function. A small T value indicates a high correlation between variables, which could lead to unstable estimates of the discriminant function coefficients. HCA was used to classify the four Nigerian cattle breeds using the nearest neighbor method (Oliveira et al. 2018).

## Results

The results presented provide a comprehensive statistical comparison of body measurements among four Nigerian cattle breeds: White Fulani (WF), Gudali (GU), Azawak (AZ), and Red Bororo (RB). The average body measurements of male and female animals are presented in Tables 1 and 2, respectively. Generally, the GU breed exhibited the highest body measurements (602.82 ± 68.86) compared to other breeds (431.80 ± 116.31 and 416.24 ± 36.63), while the AZ cattle showed the lowest body measurements (308.28 ± 49.27). In the GU and RB breeds, males had higher body measurements (602.82 ± 68.86 and 416.24 ± 36.63) than females (555.14 ± 69.42 and 387.90 ± 60.19). However, in the WF and AZ breeds, the body measurements of males (431.80 ± 116.31 and 308.28 ± 49.27), and females (435.26 ± 113.80 and 308.12 ± 45.19) were similar.

**Table 1:** The body measurements in four Nigerian male cattle breeds

Parameters	White Fulani	Gudali Cattle	Azawak	Red Bororo
Adult weight (kg)	431.80 ± 116.31a	602.82 ± 68.86b	308.28 ± 49.27c	416.24 ± 36.63a
Head width (cm)	36.32 ± 2.29a	62.70 ± 9.17b	34.96 ± 6.08a	34.54 ± 4.28a
Head length (cm)	56.42 ± 2.49a	62.88 ± 8.99b	43.28 ± 10.03c	53.04 ± 6.94d
Body length (cm)	170.20 ± 15.90a	195.84 ± 20.52b	97.76 ± 8.09c	166.10 ± 21.96a
Body width (cm)	112.78 ± 11.78a	130.22 ± 26.07b	92.18 ± 9.25c	111.46 ± 16.03a
Body depth (cm)	101.08 ± 9.53a	127.42 ± 18.62b	88.18 ± 11.28c	99.66 ± 9.63a
Chest length (cm)	33.08 ± 3.62a	55.94 ± 12.40b	33.08 ± 3.62a	31.44 ± 4.77a
Chest girth (cm)	163.42 ± 16.90a	203.78 ± 34.44b	87.98 ± 10.54c	148.18 ± 31.46d
Chest width (cm)	31.98 ± 4.82a	37.58 ± 7.13b	30.02 ± 5.73a	30.20 ± 6.23a
Chest depth (cm)	86.30 ± 10.24a	111.12 ± 15.16b	52.54 ± 7.62c	80.46 ± 16.99d
Sternum height (cm)	129.18 ± 6.06ac	145.56 ± 36.20d	112.50 ± 18.44a	120.96 ± 15.41ab
Withers height (cm)	125.76 ± 5.77a	172.66 ± 40.92b	114.44 ± 18.69c	125.32 ± 6.37a
Rump height (cm)	131.36 ± 5.68a	157.82 ± 27.43b	110.78 ± 22.28c	125.22 ± 15.42a
Rump length (cm)	43.92 ± 2.54a	58.98 ± 6.31c	40.20 ± 5.65b	39.84 ± 7.99b
Rump width (cm)	39.80 ± 3.14a	55.20 ± 10.26b	28.20 ± 6.08c	33.08 ± 8.61d
Hump length (cm)	29.02 ± 9.16a	52.00 ± 10.22c	24.56 ± 9.85b	26.86 ± 8.98ab
Hump width (cm)	14.14 ± 4.63	14.72 ± 4.32	13.46 ± 3.74	13.16 ± 4.04
Horn length (cm)	45.70 ± 11.51a	48.62 ± 9.28b	36.38 ± 6.59a	45.12 ± 11.17a
Ear length (cm)	23.30 ± 3.19a	27.12 ± 5.29b	22.74 ± 3.09a	22.84 ± 4.02a
Dewlap width (cm)	22.12 ± 5.41a	33.84 ± 10.55b	19.80 ± 5.17a	21.30 ± 4.45a
Tail length (cm)	105.34 ± 11.75ab	108.86 ± 19.74a	101.64 ± 13.08b	101.48 ± 14.31b
Cannon circumf. (cm)	18.02 ± 2.07a	18.20 ± 2.17a	16.76 ± 1.88b	18.02 ± 2.07a
Scrotal circumf.(cm)	24.76 ± 2.64a	39.16 ± 5.86b	21.68 ± 22.48c	22.48 ± 3.53c
Scrotal length (cm)	23.60 ± 3.35a	27.94 ± 3.93b	23.68 ± 3.31a	23.96 ± 3.53a

Superscript within different rows differ significantly (*P* < 0.05)

**Table 2:** The body measurements in four Nigerian female cattle breeds

Parameters	White Fulani	Gudali Cattle	Azawak	Red Bororo
Adult weight (kg)	435.26 ± 113.80a	555.14 ± 69.42b	308.12 ± 45.19c	387.90 ± 60.19d
Head width (cm)	36.14 ± 1.81a	61.02 ± 9.90b	34.78 ± 5.28a	34.60 ± 4.31a
Head length (cm)	55.64 ± 2.20a	62.18 ± 9.60b	43.62 ± 10.12c	51.06 ± 6.30d
Body length (cm)	168.64 ± 15.91ab	175.16 ± 18.23b	102.02 ± 26.97c	162.96 ± 19.34a
Body width (cm)	112.98 ± 10.68a	114.30 ± 18.87a	90.88 ± 12.28b	113.52 ± 12.75a
Body depth (cm)	101.22 ± 8.00a	101.64 ± 8.65a	92.26 ± 7.76b	99.20 ± 8.86a
Chest length (cm)	32.76 ± 3.50a	43.76 ± 13.17b	32.76 ± 3.50a	30.46 ± 5.65a
Chest girth (cm)	164.34 ± 16.44a	167.96 ± 27.15a	88.60 ± 10.33b	154.70 ± 27.02c
Chest width (cm)	32.06 ± 4.98a	33.84 ± 5.81ac	29.34 ± 6.29b	30.10 ± 6.12ab
Chest depth (cm)	86.90 ± 9.89a	93.42 ± 12.60b	50.74 ± 7.86c	82.12 ± 16.84a
Sternum height (cm)	128.94 ± 5.18ab	131.64 ± 16.24a	106.56 ± 22.50c	123.50 ± 12.84a
Withers height (cm)	125.22 ± 5.45a	133.32 ± 23.94b	113.96 ± 17.83c	122.50 ± 8.94a
Rump height (cm)	131.24 ± 5.06a	136.78 ± 17.03ab	113.14 ± 23.21c	126.38 ± 12.97a
Rump length (cm)	43.74 ± 2.41a	44.82 ± 4.22a	40.52 ± 4.22b	39.48 ± 7.72b
Rump width (cm)	38.22 ± 4.56a	42.76 ± 11.02b	27.94 ± 6.32c	34.44 ± 8.17d
Hump length (cm)	27.76 ± 7.27a	52.70 ± 10.14c	23.74 ± 9.26b	25.24 ± 7.83ab
Hump width (cm)	13.52 ± 3.97a	15.30 ± 4.84b	13.12 ± 3.36a	12.86 ± 3.46a
Horn length (cm)	49.74 ± 12.57a	49.06 ± 8.01a	37.32 ± 7.29b	48.86 ± 11.79a
Ear length (cm)	23.70 ± 3.06a	25.74 ± 5.14b	23.30 ± 3.02a	23.08 ± 4.19a
Dewlap width (cm)	22.26 ± 4.19a	33.98 ± 9.20b	20.42 ± 4.52a	22.24 ± 4.01a
Tail length (cm)	106.74 ± 10.68ab	111.12 ± 14.50a	102.08 ± 10.88b	103.58 ± 14.66b
Cannon circumference	23.68 ± 1.63a	23.28 ± 5.21a	26.90 ± 1.39b	23.73 ± 1.63a
(cm) Udder length (cm)	15.92 ± 3.70a	25.44 ± 6.63b	15.90 ± 3.21a	15.44 ± 2.92a
Udder diameter (cm)	23.32 ± 2.61a	26.86 ± 4.39b	21.28 ± 3.50c	21.62 ± 3.30c
Udder teat length (cm)	3.67 ± 1.27a	5.80 ± 2.65b	3.84 ± 1.39a	4.86 ± 1.35c

Superscript within different rows differ significantly (*P* < 0.05)

To evaluate how effectively body measurements can distinguish between cattle breeds, the CDA revealed that body measurements can effectively characterize four Nigerian cattle breeds using 13 discriminant variables in pooled animals (Tables 3 and 4). Specifically, the current study found that body measurements can discriminate the four Nigerian cattle breeds (pooled animals) into their original groups, with the following classification accuracies: WF (0.87), GU (0.99), AZ (0.98), and RB (0.70), as shown in Table 4.

**Table 3:** Selected body measurements by stepwise discriminant analysis to characterize four mixed- sex Nigerian cattle breeds*

Discriminant variable	T	Fremove	D2	λ
Head width	0.95	90.76	1.24	0.04
Body length	0.81	60.08	1.30	0.03
Chest girth	0.69	17.27	1.22	0.03
Chest length	0.80	15.45	1.30	0.03
Hump length	0.78	26.98	1.25	0.03
Head length	0.96	16.28	0.99	0.03
Hump width	0.77	14.16	1.30	0.03
Rump length	0.75	9.31	1.08	0.02
Chest depth	0.65	8.55	1.28	0.02
Body depth	0.62	8.93	1.18	0.02
Rump width	0.90	7.09	1.03	0.02
Dewlap width	0.90	4.61	1.30	0.02
Body width	0.76	4.10	1.24	0.02

T: tolerance; D2: minimum D square; λ: Wilks lambda

*Scrotal and udder measurements were excluded

**Table 4:** Percent and number of observations classified into breeds

Breed	White-Fulani (%)	Gudali-Cattle (%)	Azawak (%)	Red Bororo (%)
White Fulani	0.87 (87)	0.00 (0)	0.01 (1)	0.12 (12%)
Gudali cattle	0.01 (1%)	0.99 (99%)	0.00 (0)	0.00 (0)
Azawak	0.00 (0)	0.00 (0)	0.98 (98%)	0.02 (2%)
Red Bororo	0.27 (27%)	0.00 (0)	0.03 (3)	0.70 (70%)

N: number and percent of observations

**Fig. 3:**
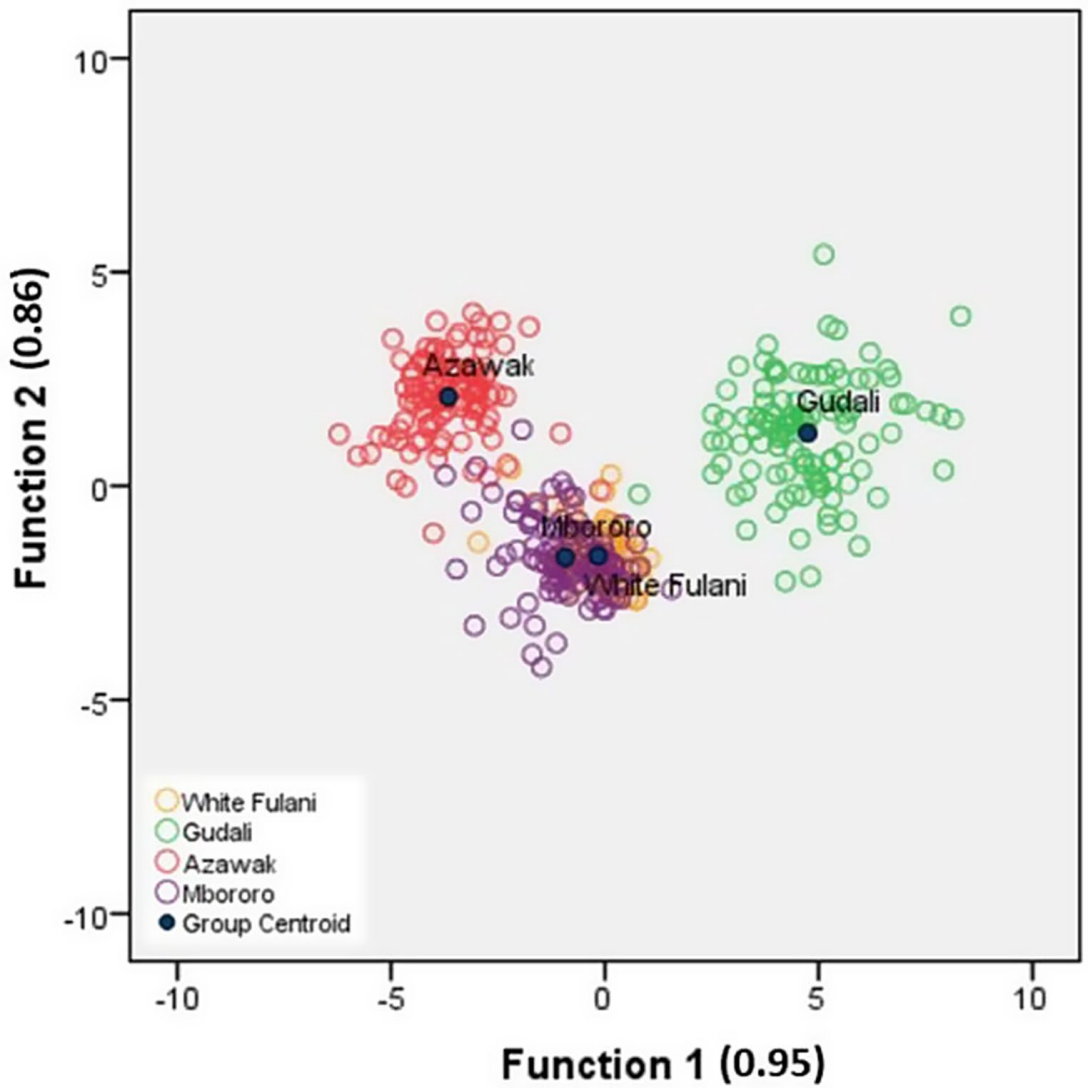
Canonical discriminant plots of body measurements in four Nigerian cattle breeds

The CDA conducted in this study reveals high canonical correlation (rc) values of 0.95 for function 1 and 0.86 for function 2, as shown in Fig. 3. In this figure, the WF and RB breeds are grouped in a similar cluster, which is distinct from the GU and AZ breeds. Additionally, the HCA analysis indicates that the closest Euclidean distance is between WF and RB (0.21), as presented in Table 5. The longest Euclidean distance is observed between GU and AZ (1.77), followed by AZ-WF (1.62), AZ-RB (1.53), GU-RB (0.72), and GU-WF (0.65).

**Table 5:** Euclidean distance in four Nigerian cattle breeds based on body measurements

Breed	White Fulani	Gudali Cattle	Azawak	Red Bororo
White Fulani	0.00	0.65	1.62	0.21
Gudali cattle		0.00	1.77	0.72
Azawak			0.00	1.53
Red Bororo				0.00

**Fig. 4:**
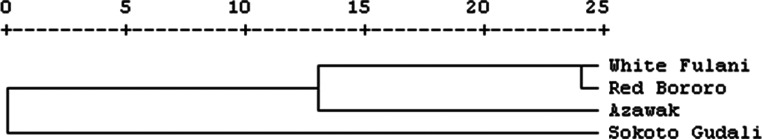
Dendogram in four Nigerian cattle breeds based on body measurements

Hence, according to Hierarchical Cluster Analysis (HCA) of body measurements, Nigerian cattle breeds are grouped into three clusters: Cluster 1 (GU), Cluster 2 (AZ), and Cluster 3 (WF and RB), as illustrated in Fig. 4.

Canonical Discriminant Analysis (CDA) in Table 6 showed that Mozambican (rc = 0.97), Eritrean (rc = 0.96), and the combined White Fulani, Muturu, and Pasundan cattle (rc = 0.95) had the highest discriminative ability. In contrast, Indian (rc = 0.48) and Algerian (rc = 0.54) cattle showed the lowest. Ethiopian cattle also performed well (rc = 0.90), highlighting the value of multivariate traits like chest girth, dewlap width, and horn length.

**Table 6:** Details of CDA in body measurements of cattle breeds

Cattle breeds	Nm	rc	Discriminant variable	Reference
Bunaji and Sokoto Gudali	10	0.58	RW, WH, HeL	Yakubu et al. (2010a, b)
Indian native	8	0.48	WH, BL, EL, TL, CG, HeL	Pundir et al. (2015)
Niger Kuri	16	0.84	-	Grema et al. (2017)
Nigerian native	19	0.85	SH, UD, HeW	Anya et al. (2018)
Eritrean native	7	0.96	CG, WH, DW, TL, EL	Goitom et al. (2019)
Ecuadorian Creole	14	0.94	-	Congo et al. (2019)
Ongole and Pasundan	6	0.81	CD, BL, CG	Putra et al. (2020)
Begaria	9	0.58	HoL, SH, EL, CG	Getachew et al. (2020)
Muturu	11	0.71	HL, BL, CG, EL	Shaibu et al. (2021)
White Fulani, Muturu, Pasundan	5	0.95	WH, BL, RL, CG	Ahmed et al. (2021)
Algerian native	13	0.54	RL, MC, CG, HeL, RW, WH	Bousbia et al. (2021)
Ethiopian native	21	0.90	CG, DW, WH, HoL, BL, RH, RW, EL,	Zegeye et al. (2021)
			CD, SC	
Ethiophian native	7	0.70	CG, CC, WH, BL, HoL, RW	Tenagne et al. (2023)
Raya	6	0.82	HoL, EL, WH, RW, CG	Mustefa et al. (2021)
Mozambican native	9	0.97	BL, HoL, RW, WH, RH,	King et al. (2022)
Harar and Ogaden	8	0.93	CC, HoL, RW, BL, EL, CG, WH	Mustefa et al. (2023)

Nm: number of variables; rc: canonical correlation of function 1

## Discussion

In the present study, 26 body measurement variables were used to characterize four Nigerian cattle breeds (Okoh et al. 2021; Babale et al. 2020), achieving a discriminant function (rc value) of 0.95 (Function 1, very high and 0.86 function 2, high) showed that these values indicate strong relationships between the discriminant functions and the breed grouping, confirming the robustness of the model. This is similar to the close rc values found in morphometric characterizations of native cattle populations in Ethiopia (0.96) and Mozambique (0.97). Similar close rc values were also reported in the morphometric characterizations of Ecuadorian Creole (0.94), Harar and Ogaden (0.93), and White Fulani, Muturu, and Pasundan (0.95) breeds, as reported by Congo et al. (2019), Mustefa et al. (2023), and Ahmed et al. (2021) in Table 6, respectively.

The high rc value in the canonical discriminant analysis (CDA) suggests that the animals were successfully characterized using the selected variables. It is also important to note that the number of discriminant variables can be influenced by the number of variables used in the CDA. Previous studies have reported that the genetic distance in Guelmoise and Fawn cattle (0.60) from Algeria (Bousbia et al. 2021) and Ethiopian native cattle from the Azebo - Alamata (0.37), Mecha - Achefer (0.58), and Tulo - Jarso (0.77) populations (Mustefa et al. 2021, 2023; Tenagne et al. 2023) were higher than that of WF-RB cattle (0.21).

The current study showed that canonical discriminant analysis (CDA), body measurements were not effective in discriminating between WF and RB breeds. This observation diverges from prior studies, wherein Canonical Discriminant Analysis (CDA) has predominantly been applied to differentiate White Fulani cattle from other morphologically distinct breeds such as Muturu and Pasundan. In those contexts, CDA effectively classified individual animals based on morphometric traits (Ahmed et al. 2021). However, the present findings indicate a substantial morphological overlap between White Fulani (WF) and Red Bororo (RB) cattle. This overlap may be attributed to shared evolutionary ancestry, gene flow, or convergent adaptation to similar environmental pressures. Such close phenotypic resemblance, as quantified through CDA, suggests that distinguishing between these two breeds based on visual appraisal or conventional field methods may be challenging and potentially unreliable (Yakubu et al. 2010a, b). Furthermore, only 70% of RB cattle could be classified into their original breed based on body measurements. Lower accuracy for RB suggests more variability or similarity with other breeds, particularly WF.

The substantial Euclidean distance observed between Gudali and Azawak cattle in morphometric analyses likely reflects pronounced morphological, ecological, and genetic divergence driven by distinct adaptive and selective pressures. Morphologically, Gudali cattle are characterized by a larger, more muscular build, with greater body weight, chest girth, and body length, consistent with their selection for meat production in humid and sub-humid agro-ecological zones (FAO 2012; Yakubu et al. 2010a, b). In contrast, Azawak cattle exhibit a leaner, taller, and more angular frame, adaptations that support endurance, mobility, and thermoregulation in arid Sahelian environments (Tawah and Rege 1996; Mekasha et al. 2008). These differences in body conformation contribute significantly to the observed variation in linear body measurements and overall morphometric profiles. Ecological specialization further reinforces this divergence, as each breed has adapted to markedly different climatic conditions, influencing traits such as skin thickness, heat tolerance, and body condition (Rege and Tawah 1999; Seifemichael et al. 2014). Additionally, the genetic differentiation between the two breeds, likely due to divergent origins and limited gene flow, has further contributed to their distinct phenotypic expressions (Hanotte et al. 2002; Mwai et al. 2015). The divergent selection objectives: meat production in Gudali versus draught utility, endurance, and heat adaptation in Azawak have led to the development of breed-specific traits that are reflected in the large inter-breed Euclidean distance, indicating the influence of both natural and artificial selection in shaping cattle phenotypes (FAO 2007; Mwacharo et al. 2006a, b).

The implication of using body measurements showed that it is reliable indicators for breed classification, especially for GU and AZ. Several studies have reported that around 70% of cattle can be classified into their original breed based on body measurements. For instance, in Mozambican native cattle, 70% of animals from the Angone (0.77) and Landim (0.73) populations (King et al. 2022), Nigerian Muturu bulls from Imo (0.75), Enugu (0.76), and Ebonyi (0.76) populations (Shaibu et al. 2021), and Ethiopian native cows from the Abergelle (0.69) and Kebri Bayah (69.70%) populations (Zegeye et al. 2021; Mustefa et al. 2023) were classified correctly. In Indonesian cattle breeds, 100% of Bali, Madura and Sasra can be classified in their original population based on 17 variables of morphometric (Adinata et al. 2023).

Interestingly, Nwachukwu et al. (2022) reported that WF and GU cattle breeds clustered similarly in a plot based on 14 microsatellite markers. In general, most African cattle exhibit genetic introgression of both Taurine and Taurindus (Taurine × Indicus) lineages, as indicated by whole genome SNP panel analysis (Decker et al. 2014). Additionally, whole genome sequencing (WGS) analysis revealed that Nigerian cattle share a close genetic relationship with Ankole cattle (Mauki et al. 2022). In WF and AZ, body measurements were nearly equal between sexes, suggesting minimal sexual dimorphism in these breeds. These findings could guide breed selection, conservation, and breeding programs in Nigeria. The high discriminative power suggests body measurements are a cost-effective method for field classification of cattle. Strong morphological distinctions exist between some breeds (e.g., GU vs. AZ), while others (e.g., WF and RB) share physical similarities, possibly due to shared ancestry, gene flow, or environmental adaptation.

To adequately classify these breeds of Nigerian cattle, future research focusing on cranial measurements can prove crucial for distinguishing Nigerian native cattle breeds, similar study on Javanese goats (Capra hircus) in Indonesia showed that cranial measurements were able to classify 100% of animals into their original breed group (Suryani et al. 2013). Hence, assessing the cranial measurements can provide further insights into their classification.

## Conclusion

This study confirms that body measurements are effective, low-cost tools for distinguishing among four indigenous Nigerian cattle breeds; White Fulani, Gudali, Azawak, and Red Bororo. Gudali exhibited the largest body dimensions, suggesting higher meat potential, while Azawak’s smaller size reflects adaptation to different production needs. Discriminant analysis showed high classification accuracy for Gudali and Azawak, highlighting the reliability of morphometric traits. However, considerable overlap between White Fulani and Red Bororo suggests a close genetic relationship, likely resulting from generations of crossbreeding, which may complicate visual identification. Overall, body measurements remain valuable for breed characterization and provide practical benefits for selective breeding, conservation, and herd management.

## Data Availability

Data supporting the findings of this study are available from the corresponding author upon reasonable request.
